# Epileptiform Neuralgia of the Fifth Nerve

**Published:** 1887-08

**Authors:** Victor Horsley


AMERICAN JOURNAL

—OF—

DENTAL SCIENCE.

Vol. xxi. Third Series—AUGUST, 1887. No. 4.

ARTICLE I.

### EPILEPTIFORM NEURALGIA OF THE FIFTH NERVE.

BY PROFESSOR VICTOR HORSLEY.

He said: It is not my intention to weary you with details of cases of neuralgia that I have treated by operation, but I wish to bring before you certain points of interest in these cases, and more especially points in regard to diagnosis, points upon which I myself desire much information; for these cases, as you may well imagine, have only come into my hands at a very late period after the onset of the mischief, the earliest 3½ years after the pain had made itself very obvious, and the latest 7 years after. In each of the cases, after the operation, there has been only one story—gratification at the relief from pain, and regret at the loss of sound and useful teeth. Of course it is on this latter point that I hope to-night to have the benefit of your wide experience given me. If we can diagnose the exact conditions under which the most inveterate form of neuralgia arises, we shall, I think, make one step forward in the right direction.

Now, if a patient comes to us and complains of infra-orbital pain, I take it we first put to ourselves the question whether the pain is caused by mischief of a central origin, and if of a peripheral origin, whether the disease is in the trunk of the nerve or in the teeth. It is not necessary for me to do more than mention the possibility of its being occasioned by intra-cranial disease. I think the general rules for the detection of intra-cranial disease suffice for its diagnosis. The remaining question, then, is whether it is possible to diagnose affections of the trunks of nerves from diseases of their terminations?

Perhaps if I just mention to you the principal points in the cases which I have treated, two of whom are in the next room, it will serve as a direction to us. The first case is a man aged 60, who suffered for seven years before I saw him. All the branches of the nerves were affected, but it was clear that the middle division of the fifth nerve was the starting point. The pain he felt and referred to was located in the centre of the malar bone, and from that point radiated all over the branches of the left fifth nerve. I operated on the infra-orbital nerve, and I was convinced by means of the electric light that I had removed the whole of the nerve from the foramen rotundum, but although he was relieved, the pain recurrrd in a few days in the palate. To make sure I cut down upon the palate at the hinder part of the alveolar border, and that operation was followed by complete relief of pain for seven months. But then the pain recurred in the inferior dental nerve, and then I performed an operation for removing a considerable piece of the inferior dental nerve. I believe that this is a secured success. It will not be sufficient for us to destroy only a part of the nerve unless we destroy the whole of that part. Now, the operation I suggest for reaching the nerve below the foramen ovale seems to me a simpler one than has been suggested recently, within the last two months, by Albrecht, of Vienna. Albrecht proposed to cut down from the zygomatic arch; but you can get at the nerve perfectly well by exposing the masseter, trephining the lower jaw, and cutting upwards into the sigmoid notch with the bone forceps, and following upon the nerve, you can trace it up to the foramen ovale. I performed this operation upon the patient about twelve months ago, and he had complete relief until this last week, he tells me he had a twinge in the palate. Whether it is going to be the usual slight recurrence I cannot say. However, he has had more than a year of complete relief.

Well, then, the next patient was also a man, aged 65. I simply performed the operation for removing the middle division. This was followed by complete relief. He remains cured so far; that was done about three months ago.

The next case is that of a matron of an infirmary who had suffered for three and a-half years; she also is relieved.

In the last case, I performed the operation a few weeks ago. It was the worst case I have ever seen. The patient, a lady, for a fortnight had not been able to feed by the mouth at all, she suffered intense pain, and the parts were a great deal swollen and glazed. The operation was of the severest kind. Although the pain was intense in the fifth nerve, it was radiated to the other branches.

Well, these are briefly the details of the cases upon which I now propose to dwell for a few moments.

I think, for the purpose of fixing the points or the problem before us, we had better consider for a moment the anatomy of the mixed nerve as a whole, and consider the question on a broad basis.

In the first place we must admit that whatever may be the ultimate physiological explanation, there must be fibres which, if damaged, disease will occur.

Next we have fibres of common sensation going to the skin. Next we have fibres going to the vessels; and lastly, we have fibres of special sensation—in these cases of course terminating in the teeth. Then mixed nerves—in fact, so far as we know, all nerves—are provided with nerves of common sensation, that is to say, there are end organs which give the trunk of the nerve common sensation. This fact is a comparatively novel one. The stem upon which the observations are based is shown here; it explains pathologically the occurrence of great pain in the periphery.

The problem for us to-night is whether we can diagnose between the inflammation of this portion of the nerve and of terminal portions. Here is the point upon which I must crave your charity, because, as I said just now, I have no experience in the initial stages of the trouble, and it is upon this special point that I ask for information. But, speaking from the other point of view, of cases as I have seen them myself, it occurred to me to ask whether we could not attempt to diagnose much in this fashion:—If the mischief affects the termination of the nerve, it will be clear that there can be no changes in the other portions of the body supplied from the other branches of that nerve, except by reflex action through the whole system, that is to say, we could not expect to get any wasting in the face by neuralgia commencing only in the teeth, or if we had, it would be very slight. If, on the other hand, you had mischief in the trunk itself, it is obvious the converse ought to be true, viz., that the trophic changes ought to be very marked. Well, I believe that is the case, that these are not accompanied by any changes through reflex mechanism. Now, what are the changes? I spoke of wasting, but there are more minute pathological changes. The patient I quoted to you as having been operated on last presented these changes in the most marked degree I have ever seen: the lower lip was greatly swollen and excessively sensitive, and the skin glazy and shiny. The same thing was noticed by Mr. Walsham in his account of two cases of the same kind. Of course one notices that the vessels may sometimes be dilated, or at others just the opposite.

Well, now, as to common sensation, it is perfectly true, as in an ordinary case of toothache you may observe, that the pain radiates not only from the parts affected but to all the branches of the fifth nerve, but I do not think it has ever been observed that there have been changes in the skin. What I would point out is this, supposing that we are dealing with pain in the upper canines; supposing, again, that pain not to be complicated with local inflammatory causes, I am not aware of cases being recorded in which the skin was hyperæsthetic or anæsthetic at a distance so far as I know. Well, this is the case when the disease is in the extreme trunk of the nerve.

Sometimes you get a case in which, if you gently rub something over the skin, it gives extreme pain, whereas, if you press it tightly there is no pain at all; that is well known, but it has never met with any explanation. That is a very striking form of hyperæsthesia, and it is one which I believe is only connected with those cases where the diseases is in the trunk of the nerve.

Lastly we come to nerves of special sensation. In the case of teeth, of course we have hyperæsthesia in varying degrees. I would ask for information on the point, whether in those cases where pain is distinctly removed by extraction of the teeth, and then observed to recur in the next tooth, whether there was any difference in the patient's feelings; I mean to say, was it an exact repetition of the original feeling, first, when the tooth is pressed upon, beginning slightly and becoming intense, did the whole process go through the next tooth? Because, *a priori*, it would follow that you are dealing with a case of local affection, and perhaps it would be good policy to go on extracting. But, as I say, that is one point upon which I desire very greatly to be informed.

Well, now, suppose we take up the character of the pain itself and analyse it as a means of diagnosing these two conditions slightly more closely. Well, I think one generalization should be considered, viz., the place or origin of the pain—where did the pain first arise?

In the first case I told you of the pain began in the bone. I find in the majority of cases the patient says it begins in the teeth, they also have it in the bone and also in the skin; of course observations of this kind are calculated to help. If the patient states distinctly that the pain begins in the bone, and from there goes to the teeth, it should tell us that the origin is in the trunk of the nerve, and that after extracting one or two teeth we should stop and go to the trunk of the nerve.

Then, again, if the pain commences very distinctly in the skin, and subsequently only affects the teeth, it would point to the mischief beginning in the trunk. Finally, if it is referred by the patient to the teeth, it might begin there, but it might not. It may be only a question of referring the pain wrongly, just in the same way that we refer pain to the little finger when it comes from the elbow. Then comes the next point, the *mode* of its origin. I suppose it is within the experience of all here that it is impossible always to find from the patient what was the original cause of the mischief. One will say it is a bad tooth, another mental worry, another cold, another injury, and so on. I have not been able to find out any common mode of origin. But, there again, my experience as a surgeon is extremely limited, and on this point I desire further information, but I may say, if the pain occurs gradually, and there are teeth in the neighbourhood of the seat of the origin of the pain as described by the patient, well then, I suppose we may say the teeth are the primary source of the mischief. If the pain occurs suddenly we may say it is in the trunk.

Well, we take another generalization concerning the pain and discuss its character. If it is of a constant character, it would seem to have in most cases a peripheral origin; if it is intermittent, it would seem to be in the trunk, but in a very severe case you may get these points confused. In the last case I quoted, the pain was distinctly intermittent. There were periods of complete relief from neuralgic pain.

Now, fourthly, with regard to excitant causes. Can pain be brought on—in other words, what is the cause of pain? If you find that pain is brought on most commonly by movement, it would seem to be characteristic of affection of the trunk of the nerve. The patients all state that movement of the jaw brings on pain; in fact, on one occasion I secured rest to a patient by tying up the jaw. It is perfectly obvious to any of us that the inferior dental nerve is pulled upon in opening the jaw to eat or speak, and this possibly will bring on pain. I am not aware that the same importance is to be attached to movement in ordinary dental irritation.

Well, as to the question whether different re-agents excite it or not, I think we can gain very little information. The same patient will tell you that cold brings it on, and another day that heat does so, so that there is no hard and fast line to be laid down on that point.

Fifthly, I think the extent of the pain is one of the most important points. If, in addition to the fact that you have severe pain in the distribution of the nerve you have distinctly tender spots along the branches of the nerve, you have an indication that the whole nerve is in a state of hyperæsthesia, and the pain is primarily due to inflammation.

Finally, gentlemen, a few remarks concerning treatment. I have endeavoured to lay before you very briefly and sketchily the outlines upon which we can discuss the origin of the pain, which is a practical matter to be dealt with. Now, as regards treatment in those cases in which extraction has not been proved to be of final service. All those cases I have treated by drugs, such as opium (of which, in one case, I gave 30 grs. a day, and in another 23 grs. a day),. &c. All these narcotics have ultimately failed; they have relieved the pain for a time, but then made it much worse. The very small experience I have had has shown me that croton-chloral produced more effect than any other drug taken internally. Then, with regard to local administration, we have seen various injections hypodermically made. Cocaine always seems to fail, and necessarily the introduction of the needle brings on a paroxysm of pain. Large doses of morphia may be given witnout any relief of pain. Some patients say it makes the pain worse. There seems to be some action on the nerve tissues, which is not altogether favourable.

With regard to surgical treatment of the nerve, there is no doubt that nerve stretching should not be performed upon the branches of the fifth nerve, because it involves just as many risks as cutting out the nerve, and the pain invariably recurs. Neurectomy, on the other hand, is generally successful. As to details in carrying out surgical treatment, of course I must not occupy your time here, but allow me to say, from seeing the operation performed under different circumstances, that I do believe that success is due to the efforts one makes to obtain primary union—this always supposing that the whole nerve, or a considerable portion of the whole nerve, has been completely removed.

Lastly, as regards the effect of neurectomy—the general effect on the parts and the general effect on the patient—I have not seen any bad effect follow. Of course, it was to be feared that by removing the nerve one might interfere with the nutritive condition, but as a matter of fact the areas which the nerves supply are remarkably limited, the area of the infra-orbital nerve are extremely small, and then again, the nerves which are most affected fortunately do not supply the most important parts. It is the nerve supplying the cheek and the lips; fortunately the nerve supplying the eye is rarely the seat of the mischief, and hence we see the possibility of escaping trophic changes; but I have really come to ask for information and not to give it. And the information I wish for is with regard to the character of the pain, the seat of the disturbance, the mode of its onset, the cause of its excitement or origin, and the possibility of the affection of other branches of the same nerve.

If these points relating to the earlier stages of the disease are cleared up by the very wide experience of the members of this Society, I think I may say we shall make a very great step forward in the right direction.

After some observations by Mr. S. J. Hutchinson, Mr. Betts, and Mr. Ashley Barrett,

The President said: There is one great puzzle to me about these neuralgia cases, and one to which I have never been able to formulate any explanation at all—I hope Professor Horsley may be able to throw some light upon it—that is the great relief of pain which is often given by doing the wrong thing; by doing the thing which you are told by subsequent experience of the case cannot have gone to the root of the evil. For instance, you cut the inferior dental nerve; well, it will very likely stop all the pain for a few weeks, but often you find it comes back as badly as ever. You clearly in your first operation and in your second operation had not got to the site of the trouble; and yet, somehow or other, you have altered the nutritive conditions, and have done something which, for the time being, has arrested the pain. I have in my recollection a case in which the nerve was first divided at the mental foramen, giving the patient great comfort for a time: he could eat, shave, or talk without any unpleasant sensations; but the pain came back. The nerve was then drilled down upon in the canal; that set him perfectly right for eight months. On the recurrence of the trouble, the operation was repeated, and the drill was run down upon the nerve in the same place. But it did no good. Subsequently the patient went to Mr. Durham, who dissected back, within the mouth, to the orifice of the dental canal, and stretched the nerve severely. This did good for the time. But the point which I should like to press upon Professor Horsley is, why the doing of ineffectual operations which do not reach the seat of the disease so often seem to do good, when subsequent knowledge shows that it could not have got to the site of the evil?

There is one other point, and that is the trophic alterations that are to be seen in these cases, and which Professor Horsley lays great stress upon, as showing the lesion to be in the nerve trunk. I have myself seen three cases of great severity in which the trophic lesions, if they existed at all, were so inconspicuous as to escape observation. One of these were treated electrically with some success.

Mr. Henri Weiss mentioned a case of peripheral neuralgia, in which a man dreaded any pressure on his face. The attacks would not come on from either heat or cold, but by pressure, and more particularly by pulling the moustache.

Mr. J. Smith Turner said Professor Horsley asks for further information as to the peculiarity of the special sensibility which occurs in patients who suffer from this disease, and that is that the skin is exceedingly sensitive to the soft touch, but that by hard pressure the same sensibility is not produced. It seems to me that this sensibility is a good deal the same which the eye experiences on being handled by the skillful or unskilful operator, as the case may be. The expert oculist handles the eye in a manner which seems to us almost brutal, but causes no pain; the timid operator approaches the eye gently, and produces an amount of pain which makes the patient shrink from his touch. I have noticed cases in which patients do shrink when the skin is slightly touched in the neighbourhood of the pain, but if you press hardly the pain is not felt. It seems to me that pressure obtunds pain. In the experiments which have been carried out with reference to this nerve stretching theory, there has been, I think, a good deal of what is called nerve pinching; and this nerve pinching has destroyed sensibility in some cases and effected the very same thing as nerve stretching. We have many obscure conditions of pain in the teeth which render peripheral diagnosis sometimes difficult. I have now a case in my recollection which was brought before the attention of the profession—I think it was before the Odontological Society was established—in which intense pain was suffered under movement; when the patient was at rest there was no pain, comparatively speaking. A tooth was removed, and on examination it was found that there was a detached piece of secondary dentine in the canal of the tooth, which moved about as the patient moved, and produced pain. Pain was also produced by the pressure of putting the head on the pillow. With regard to end organs, if my memory serves me, attention was drawn to the subject in a paper by Professor Marshall some three to five years ago, and a diagram was drawn of the nerves—portions of nerves which seem to spring out from the trunk on the main nerve. I believe Professor Horsley was present, and referred to them as the *nervi nervorum*. These filaments, which are nerves of sensation supplying the nerves themselves, are the subject of considerable irritation; and if we have these nerves springing from the inferior dental nerve after it enters the bony canal, we may thus be able in some measure to account for the cessation of pain when we do the wrong thing, and it returns again when the nerve assumes its wonted condition.

Mr. Stocken: Some time ago I brought before the Society two cases which came under my observation. Perhaps I may be permitted to mention them again, as some little light may be thrown on the discussion thereby. One was a very severe form of neuralgia. The lady was unable to speak without great distortion of the features; the tongue was swollen, the eye-lid drooped, and the saliva was constantly running out of the mouth. She had tried several remedies, some homœopathic ones, but got no relief. The peculiarity was, that as soon as she assumed a recumbent position she obtained relief. When I saw she had lost seven teeth, as a forlorn hope I recommended her taking gelsemium 15 minims and aconite 5 minims alternately. This was seven years ago, and she has been relieved from pain ever since. The other case, that of a lady who has since died, was of a similar character. She was suffering almost as acutely and had distortion of the features. I could only discover a very small abrasion on the canine tooth. I removed it, crushed it, and examined the pulp under the microscope, and found that the whole mass was an agglomeration of calcified bodies. She was relieved for a time, but the pain returned as badly as before, and when I last heard of her, she was suffering so much that she was unable to come to London.

Mr. Storer Bennett: With regard to the question, Do we know of any cases of trophic lesions to distant parts distinctly referable to the teeth? I have a very distant recollection of trophic lesion in a girl, the lesion being an ulcer of the cornea of many months' duration. It had been treated in the ordinary way by several surgeons without any success whatever. The case was seen by Mr. Nunn, of the Middlesex Hospital, who sent her to me. He said he was quite sure that on the eruption of the canine tooth the trouble would disappear. As a matter of fact, when the canine tooth in the course of time was erupted, the ulcer of the cornea did disappear.

You mentioned, sir, the cases when wrong treatment of injured nerves produces temporary relief. I have in my recollection the case of a patient 40 years of age, looking many years older, who was perfectly edentulous in the upper jaw; all the teeth were taken out for supposed neuralgia, but when they were all taken out it was obvious that the pain was not due to the teeth. It was then suggested that something in the bone might be the cause, and the bone was drilled into, but without any good result. Then Mr. Henry Morris cut down upon the infra-orbital nerve and stretched it. The pain returning, he next cut into the infra-orbital canal and removed the nerve up to the fissure. On this nerve he discovered some little bodies, similar to those drawn by Professor Horsley, which seemed distinctly to be some little fibrous nodules. The operation ave relief.

Professor V. Horsley, replying, said, with respect to the undoubted fact of operation upon the extremity of the nerve producing relief for the time, and yet not getting at the root of the mischief: Well, it is obvious, theoretically, we may have been creating violent counter-irritation at the end of the nerve, and I should think that the category of cases belongs to Professor Gros's instances relieved by drilling. Then, again, of course it may be, as Mr. Hutchinson suggested, in these cases the mischief begins peripherally, and extending towards the centre you may have a creeping peripheral neuritis, and therefore there is no reason at all why it should not affect the branches of the fifth nerve. However, that is one point to be solved.

You said, sir, we might have trophic lesions absent in some severe cases. It was so in my own. To that I have no explanation to offer. It is of great interest to me to understand that the pain may be severe for a few minutes, then suddenly disappear, then occur again in an epileptiform manner. This complicated the diagnosis considerably. Mr. Turner also referred to peripheral disease of the teeth where the interior of the canal was affected, and of course removal cured the patient.

Then, as regards the point raised with reference te the wisdom teeth, that is a matter upon which I came to ask rather than give information. Then the cases of Mr. Stocken are extremely valuable, and it is of great interest to me to know that such cases, and after so long a period as nine years' suffering, can be treated by drugs; it gives one great hope that operation may become unnecessary. With respect to Mr. Storer Bennett's case of trophic ulcer of the cornea, I am aware of many cases published of disturbances of parts, not necessarily trophic, produced by peripheral irritation. The question I was really asking with regard to cases of trophic changes was, under what circumstances do these occur most commonly? If you have a case of severe neuralgia, so-called neuralgia, if you have very distinct disease, do you have trophic disturbances? I suspect it would be answered in the negative.

Then again, with reference to painful spots, the pain is referred to the periphery. I know there is no very great difference between slight tenderness and distinctly local pain that you get in these cases which is apparently due to the condition along the branches of the nerve.

Well now, with regard to the changes in the nerves I have removed, they all present great overgrowth of epineurium. The course of the nerve in the bone I believe to be the fountain of the mischief, and it was for that purpose I proposed that the nerve should be divided above the bone.

As to the discussion of the possible pathology, I do not know anything, and, as I said before, it is upon that point I seek information.

As regards the easy diagnosis of so-called tender teeth, I am not quite sure on that point, because a gentleman consulted me who had a first bicuspid removed in the lower jaw which made the pain worse in the next tooth. I think, unless we adopt the theory of a creeping neuritis, we cannot admit that this was a typical case of disease beginning in the periphery. I think, sir, that that concludes the remarks that I would venture to make; as I said before, I did not come here to teach, but to learn. I would just add one word, viz., that these operations in general are not so severe as they sound. Here, sir, is a photograph of my second case, the removal of the whole division of the fifth nerve, taken five days after the operation. I admit, of course, that if proper precautions were not taken these operations would be very formidable, but with the teaching of Sir Joseph Lister and others, I do not think we need fear very much.—*The London Dental Record.*

